# Study on Multi-Agent Evolutionary Game of Emergency Management of Public Health Emergencies Based on Dynamic Rewards and Punishments

**DOI:** 10.3390/ijerph18168278

**Published:** 2021-08-05

**Authors:** Ruguo Fan, Yibo Wang, Jinchai Lin

**Affiliations:** School of Economics and Management, Wuhan University, Wuhan 430072, China; rgfan@whu.edu.cn (R.F.); jc_lin@whu.edu.cn (J.L.)

**Keywords:** COVID-19, regulation, evolutionary game, subsidy and punishment mechanism

## Abstract

In the context of public health emergency management, it is worth studying ways to mobilize the enthusiasm of government, community, and residents. This paper adopts the method of combining evolutionary game and system dynamics to conduct a theoretical modeling and simulation analysis on the interactions of the behavioral strategies of the three participants. In response to opportunistic behavior and inadequate supervision in the static reward and punishment mechanism, we introduced a dynamic reward and punishment mechanism that considers changes in the social environment and the situation of epidemic prevention and control. This paper proves that the dynamic reward and punishment mechanism can effectively suppress the fluctuation problem in the evolutionary game process under static scenarios and achieve better supervision results through scenario analysis and simulation experiments.

## 1. Introduction

Since the first case of a person infected with the new coronavirus (COVID-19) was discovered in December 2019, the epidemic caused by the virus has spread rapidly around the world, causing a global health crisis. As of 1 January 2021, about 82 million people have been infected with COVID-19 worldwide, of which about 1.8 million people have died [1]. This COVID-19 outbreak is a severe challenge to the governance systems of all affected countries and cities. China, as a country that was affected by the COVID-19 epidemic earlier, has accumulated a lot of effective experience in responding to major public health emergencies, which has attracted the attention of scholars. At present, the epidemic has been controlled in China as a whole [2], and normal life and production have resumed in most areas. Although there are still small-scale outbreaks of the epidemic in some areas, they have been quickly suppressed and have not cause further spread.

Analyzing the experience of China’s national defense control of the epidemic shows that the community played an important role in fighting the epidemic and cutting off the chain of virus transmission, especially during the severe period of the epidemic situation in China. When guiding the prevention and control of the new crown pneumonia epidemic, Chinese President Xi Jinping emphasized that the community is the first line of joint prevention and control of the epidemic, as well as the most effective line of defense for external input cases and internal cases spread. The National Health and Construction Commission of China issued the “Notice on Strengthening Community Prevention and Control of Pneumonia Epidemics Caused by New Coronavirus Infection” [3] as soon as possible. China’s Ministry of Civil Affairs and the National Health and Construction Commission jointly issued the “Precise and refined guidance plan for epidemic prevention and control of COVID-19 and service work in community” [4]. All governmental measures require all regions to pay close attention to the community as the basic link to prevent and control the epidemic, and provide support and guarantee for improving the community prevention and control system through corresponding systems and policies.

Apart from the community, it is essential for residents to cooperate with the epidemic prevention work through their behavioral restraints to control the epidemic situation. At the beginning of the outbreak, the State Council of PRC issued the “Guidelines for prevention and health protection against 2019 Novel Coronavirus Pneumonia in public places” [5], carrying out publicity and education for residents about sanitary protection measures in public places through various channels. The government subsequently issued several policy documents, which gave more detailed guidance on health precautions in all aspects of residents’ lives. The residents’ initiatives to cooperate with epidemic prevention and control have achieved good results, such as wearing masks, paying attention to personal hygiene, avoiding unnecessary gatherings, reporting fever symptoms, etc.

In managing public health emergencies, there are three main bodies: The governments, communities, and residents. However, conflicts of interest exist between these subjects. Government departments aim to maintain social stability and improve overall social welfare. The community and residents may show a lower willingness to participate due to cost reasons or fluke mentality. In order to improve the government’s leading role, as well as the enthusiasm of the community and residents to cooperate, it is important to analyze the interaction of each agent’s behavior strategy and explore the key factors that affect each’s strategy.

Exploring the root causes of the conflicts of interest among the participating subjects reveals that the main reason for the poor governance of public health emergencies is due to various opportunistic behaviors and inadequate supervision. Therefore, the purpose of this article is to establish a theoretical model of the interaction of participants’ behavior and strategy and discuss how to coordinate the inconsistent interests of various subjects by restricting opportunistic behavior and improving supervision efficiency, so as to provide some insights for improving the governance effect of public health emergencies.

This paper establishes a system dynamics model of the tripartite evolutionary game of static and dynamic reward and punishment mechanism with a background of the management of public health emergencies. Based on numerical analysis, we conduct further simulation experiments to study the main factors influencing the evolution of participant behavior strategies. Finally, we put forward some policy recommendations for optimizing the management of public health emergencies. The rest of this paper is organized as follows: Section 2 introduces the literature related to our research. In Section 3, we describe the problems to be studied and model assumptions, then show the process of evolutionary game modeling and equilibrium analysis. In Section 4, we carry out the system dynamics modeling of the tripartite evolutionary game process. Through the simulation of different scenarios, we analyzed the impact of key exogenous variables on the game equilibrium. Finally, in Section 5, we summarize the main conclusions, policy recommendations, and limitations of this article.

## 2. Literature Review

The community is the basic unit of grassroots governance, and the governance of urban and rural communities is related to the implementation of the country’s major policies. Based on the community governance framework, S Bowles and H Gintis affirmed the important role of the community in national governance through the study of examples and experimental evidence. They believed that the community could solve the problems caused by market failure or government failure [6]. Research by Parag Y et al. [7] shows that communities are important promoters of bottom-up policy changes. They can provide political space and support for new government policies and plans. In China, although the concept of community governance is somewhat similar to governance in other regions, it has unique characteristics: the community has a clear institutional identity, a clear geographic scope and population, and is equipped with dedicated staff. To a certain extent, it is similar to a new form of local government [8]. In recent years, the Chinese government has attached great importance to community governance and related work. In the 19th CPC National Congress report in 2017, it was clearly stated that “China must strengthen the construction of community governance system and promote the downward shift of the social governance center to the grassroots level” [9]. China’s “Government Work Report” [10] in 2019 also emphasized the importance of “building a new pattern of urban and rural community governance.”

The community plays an essential role in the emergency management of major public health emergencies. For this COVID-19 epidemic, Marston C et al. [11] believe that community participation is essential for controlling and responding to the COVID-19 epidemic. Whether cooperating with the lockdown policy or providing necessary support to residents, the community plays an essential role in preventing and controlling the epidemic. Blendon R J et al. [12] conducted a national survey to understand residents’ attitudes towards community prevention and control measures. The results showed that most interviewees agreed to cooperate with community prevention and control measures. However, the effectiveness of residents’ cooperation depends on the level of pre-preparation, and many American residents have not made any preparations for public health emergencies. Therefore, the government and communities need to plan for prevention and control carefully and mobilize residents to actively prevent and control the epidemic through public health education and other methods. Not only do the community play a role in allocating materials and helping residents, the prevention and control methods and effects of the community will also affect the epidemic situation, because infectious diseases often show the characteristics of community transmission [13,14,15].

In addition to the prevention and control measures of the government and the community, the residents’ awareness of epidemic prevention is also critical. During the epidemic, residents took the initiative to wear masks [16,17,18], maintain social distancing [19,20,21], and reduce unnecessary travel [22,23]. Such measures have been proven to have a positive impact on the control of the epidemic. However, residents, who take active epidemic prevention and control measures or cooperate with the government and the community’s epidemic prevention and control policies, will increase living costs or reduce the convenience of life. The higher cost of cooperation is the main reason for the lower willingness of this group to cooperate.

In previous studies, researchers mainly focused on analyzing the advantages of community governance or the importance of residents’ participation and cooperation separately, in the context of public health emergencies. Few studies have analyzed the conflicts of interest, difficulties in participation, and the stable equilibrium of the participants from the perspective of the behavioral and strategic interactions of the participating subjects involved in the emergency management of public health emergencies. To fill this gap, this article will conduct further research through evolutionary game theory. The evolutionary game has been extended and applied by scholars in environmental science and resource utilization [24,25,26], preventive medicine and hygiene [27,28,29], energy economy and sustainable development [30,31,32], and other fields; this is a proven method for analyzing the interaction of multi-agent behavior strategies. Evolutionary game theory is usually integrated with system dynamics to describe the evolution process of each subject’s complex behavioral decision-making and the complex logical relationship between various influencing factors. System dynamics is a comprehensive method based on system theory, feedback theory, and information theory, with the help of computer simulation technology, to understand and solve system problems. Due to the advantages of system dynamics in the analysis of complex system evolution, scholars often combine it with evolutionary game theory to model and simulate complex problems in the real world [24,30,33,34].

Inspired by previous work, this research aims to study the emergency management of public health emergencies from the perspective of strategic interaction, which considers three participants (government, community, and residents), and combines evolutionary game and system dynamics methods. The article has obtained reached enlightening conclusions and proposed corresponding emergency management policy recommendations for public health emergencies through in-depth scenario simulation analysis.

## 3. Three-Party Evolutionary Game Modeling and Stability Strategy Analysis

### 3.1. Parameter Assumptions and Modeling

The participants in the game are the government, communities, and residents, and each group is risk-neutral bounded rationality subject to maximize their interests. During the epidemic period, the government’s strategic actions include “high-intensity regulation” and “low-intensity regulation”. The strategic behaviors of the communities include “strengthen community prevention and control” and “do not strengthen community prevention and control”. Residents’ strategic behaviors include “cooperate with epidemic prevention and control work” and “do not cooperate with the epidemic prevention and control work”. The concept of “community” mentioned in our article refers to the “community” under China’s governance structure, a grassroots mass autonomous organization for Chinese residents. The community plays a role in the management of public affairs within the jurisdiction. The government has a management obligation to the community and resident, while the community has a management obligation to the resident of its jurisdiction.

To facilitate analysis and solution, we assumed that the government strictly implements all policies once it chooses “high-intensity regulation”. The government can observe whether communities and residents cooperate with epidemic prevention and control, and punish communities or residents who do not cooperate. In this study, we do not consider rent-seeking and collusion behaviors among various subjects. The specific assumptions are as follows:

1. Participants of this evolutionary game: The article selects the main relevant interest groups related to prevention and control measures in the epidemic: government, community, and residents. All three types of participants have bounded rationality.
2. The government’s game strategy: The government has two strategies under this game model. The first strategy is a high-intensity regulatory strategy. When the government adopts a high-intensity epidemic prevention and control strategy, it brings health benefits to society. At the same time, the government guides communities and residents to strengthen epidemic prevention and control through subsidies, penalties, personnel assistance, and epidemic prevention propaganda. The second strategy is a low-intensity regulatory strategy. The government’s epidemic prevention and control efforts are only maintained at a basic level. No additional prevention and control policies are adopted to mobilize the enthusiasm of communities and residents for prevention and control. Therefore, assuming that the government behavior strategy space is $S_{Government}$ = ($X_{1}$: High-intensity prevention and control, $X_{2}$: Low-intensity prevention and control), the two strategy probabilities are x and (1−x), respectively.
3. The game strategy of the community: The community has two strategies under this game model. The first strategy is to strengthen prevention and control in the community. The community adopts stronger epidemic prevention and control measures. It provides residents with a safer and healthy environment by blocking the community, restricting travel time, and disinfecting the area under the community’s jurisdiction. The second strategy is not to strengthen community prevention and control. The community does not make additional responses to the epidemic situation, and residents will not receive additional health benefits. Therefore, assuming that the community behavior strategy space is $S_{Community}$ = ($Y_{1}$: Strengthen community prevention and control, $Y_{2}$: Do not strengthen community prevention and control), the two strategy probabilities are y and (1−y), respectively.
4. The game strategy of the resident: Residents have two strategies under this game model. The first strategy is to cooperate with epidemic prevention and control work, which means that residents cooperate with the society’s epidemic prevention and control policies, and actively improve their health awareness. The second strategy is not to cooperate with epidemic prevention and control work. Residents do not cooperate with the social epidemic prevention and control policies due to the cost of cooperation or lack of health awareness, making it difficult for the government and the community to carry out epidemic prevention work. Therefore, assuming that the resident’s behavior strategy space is $S_{Residents}$ = ($Z_{1}$: Cooperate with epidemic prevention work, $Z_{2}$: Do not cooperate with epidemic prevention work), the two strategy probabilities are z and (1−z), respectively.
5. The parameters of the game model: Under each game strategy combination, according to the cost, income, and loss of the government, community, and residents, the parameter settings are shown in Table 1.
6. Parameter constraint assumptions: Based on the actual situation of epidemic prevention and control, we make the following assumptions about the parameter constraints in the payment matrix. When the government chooses high-intensity regulation, the cost of the strategy is greater than when it adopts low-intensity regulation, that is $C_{X}^{H}>C_{X}^{L}$. The government’s prevention and control cost should be greater than the prevention and control cost of the community and residents, that is $C_{X}>C_{Y}>C_{Z}$. The prevention and control costs of communities and residents are greater than the government’s punishment for them. Otherwise, the communities and residents will not be motivated to choose negative prevention and control measures, namely $C_{Y}>F_{Y}$, $C_{Z}>F_{Z}$. Similarly, the reputation loss of governments and communities should be less than their prevention and control costs, namely $C_{X}>L_{X}$, $C_{Y}>L_{Y}$. The health benefits brought to residents by the government’s active prevention and control should be higher than the health benefits brought to residents by the community’s active prevention and control, namely $H_{Z}^{X}>H_{Z}^{Y}$.

According to the model assumptions and the game parameters shown in Table 1, the benefits and payments of the three-party game subject of the government, communities, and residents are shown in Table 2.

### 3.2. Analysis of the Evolutionary Stability Strategy of Each Participant

#### 3.2.1. Analysis of the Government’s Evolutionary Stability Strategy

According to the assumptions in the previous section, the probability of the government choosing $X_{1}$ (High-intensity regulation) strategy is x, and the probability of choosing $X_{2}$ (Low-intensity regulation) strategy is (1−x). $U_{X_{1}}$ and $U_{X_{2}}$ are used to represent the expected benefit of the group that the government chooses $X_{1}$ and $X_{2}$, respectively, and represent the overall expected benefit of the governments, so:

The expected benefits when the government chooses a high-intensity regulatory strategy are:

(1) $$\begin{array}{l} U_{X_{1}}=yz(-C_{X}^{H}+R_{X}+R_{Y}+R_{Z}-G_{Y}^{X}-S_{Y}^{X})+y(1-z)(-C_{X}^{H}+R_{X}+R_{Y}-G_{Y}^{X}-C_{X}^{Z}-S_{Y}^{X})\\ +(1-y)z(-C_{X}^{H}+R_{X}+R_{Z}-S_{Y}^{X})+(1-y)(1-z)(-C_{X}^{H}+R_{X}-C_{X}^{Z}-S_{Y}^{X}) \end{array}$$

The expected benefits when the government chooses a low-intensity regulatory strategy are:

(2) $$\begin{array}{l} U_{X_{2}}=yz(-C_{X}^{L}+R_{Y}+R_{Z}-L_{X})+y(1-z)(-C_{X}^{L}+R_{Y}-L_{X})+(1-y)z(-C_{X}^{L}+R_{Z}-L_{X})\\ +(1-y)(1-z)(-C_{X}^{L}-L_{X}) \end{array}$$

According to Equations (1) and (2), the overall expected benefits of the two government’s decisions are:

(3) $$U_{X}=xU_{X_{1}}+(1-x)U_{X_{2}}$$

According to Equation (3), the replicator dynamics equation of government groups can be calculated as:

(4) $$\begin{array}{cc} F(x) & =\frac{dx}{dt}=x(1-x)(U_{X_{1}}-U_{X_{2}})\\ & =x(1-x)[y(-G_{Y}^{X})+z(C_{X}^{Z})-C_{X}^{H}+C_{X}^{L}+L_{X}+R_{X}-C_{X}^{Z}-S_{Y}^{X}] \end{array}$$

To further analyze the impact of the size of government groups with different strategies on the stable equilibrium of government’s strategy evolution, we can obtain the derivative of the replicator dynamics equation with respect to *x*:

(5) $$\frac{dF(x)}{dx}=(1-2x)[y(-G_{Y}^{X})+z(C_{X}^{Z})-C_{X}^{H}+C_{X}^{L}+L_{X}+R_{X}-C_{X}^{Z}-S_{Y}^{X}]$$

For governments, we can make the following summary according to Equation (5):

(1) When $\frac{z(C_{X}^{Z})-C_{X}^{H}+C_{X}^{L}+L_{X}+R_{X}-C_{X}^{Z}-S_{Y}^{X}}{G_{Y}^{X}}<y\leq 1$, $\frac{dF(x)}{dx}{|}_{x=1}>0$ and $\frac{dF(x)}{dx}{|}_{x=0}<0$, the evolutionary stable strategy (ESS) of the government group is $x^{*}=0$. In this scenario, when the proportion of communities that choose “strengthen community prevention and control” strategy y is high, the government group tends to choose the “low-intensity regulation strategy” $X_{2}$.

(2) When $y=\frac{z(C_{X}^{Z})-C_{X}^{H}+C_{X}^{L}+L_{X}+R_{X}-C_{X}^{Z}-S_{Y}^{X}}{G_{Y}^{X}}$, F(x)≡0, in this scenario, any proportion x that the government chooses “high-intensity regulatory” strategy is an evolutionary stable strategy.

(3) When $0\leq y<\frac{z(C_{X}^{Z})-C_{X}^{H}+C_{X}^{L}+L_{X}+R_{X}-C_{X}^{Z}-S_{Y}^{X}}{G_{Y}^{X}}$, $\frac{dF(x)}{dx}{|}_{x=0}>0$ and $\frac{dF(x)}{dx}{|}_{x=1}<0$, the evolutionary stability strategy (ESS) of the government group is $x^{*}=1$. In this scenario, when the proportion of communities that choose “strengthen community prevention and control” strategy y is low, the government group tends to choose the “high-intensity regulation” strategy $X_{1}$.

**Figure 1:**
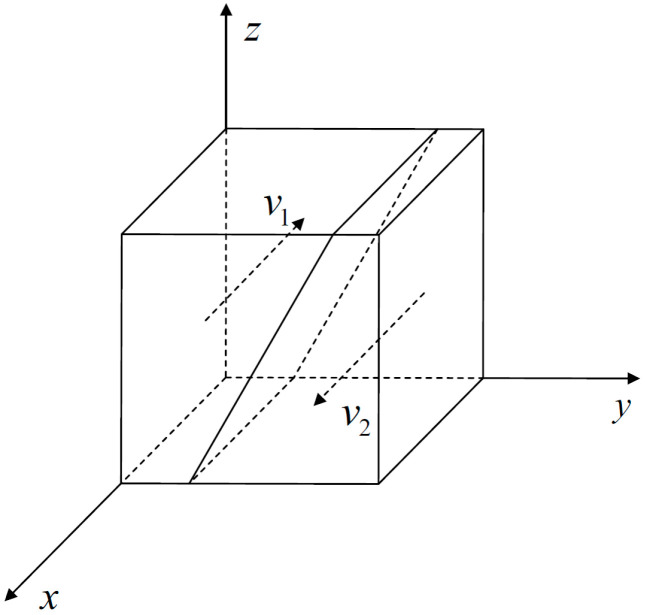
Dynamic replication phase diagram of the evolution of government decision-making.

Based on the above analysis, we can draw a dynamic replication phase diagram of government groups, as shown in Figure 1. We can see from the figure that the feasible region of each game participant is divided into two adjacent regions by the intersection space of y and z. When the feasible region is located in space $v_{1}$, x converges to $x^{*}=1$, and it is the optimal decision for the government group to adopt a high-intensity regulation strategy. When the feasible region is located in space $v_{2}$, x converges to $x^{*}=0$, and it is the optimal decision for the government group to adopt a low-intensity regulation strategy.

#### 3.2.2. Analysis of the Community’s Evolutionary Stability Strategy

According to the assumptions in the previous section, the probability of the community choosing $Y_{1}$ (strengthen community prevention and control) strategy is y, and the probability of choosing $Y_{2}$ (Do not strengthen community prevention and control) strategy is (1−y). $U_{Y_{1}}$ and $U_{Y_{2}}$ are used to represent the expected benefit of the group that the community chooses $Y_{1}$ and $Y_{2}$, respectively, and represent the overall expected benefit of the community, so:

The expected benefits when the community chooses “strengthen community prevention and control” strategy are

(6) $$\begin{array}{l} U_{Y_{1}}=xz(-C_{Y}+G_{Y}^{X}+S_{Y}^{X})+x(1-z)(-C_{Y}+G_{Y}^{X}-C_{Y}^{Z}+S_{Y}^{X})+(1-x)z(-C_{Y})\\ +(1-x)(1-z)(-C_{Y}-C_{Y}^{Z}) \end{array}$$

The expected benefits when the community chooses “do not strengthen community prevention and control” strategy are

(7) $$U_{Y_{2}}=xz(-F_{Y}+S_{Y}^{X}-L_{Y})+x(1-z)(-F_{Y}+S_{Y}^{X}-L_{Y})+(1-x)z(-L_{Y})+(1-x)(1-z)(-L_{Y})$$

According to Equations (6) and (7), the overall expected benefits of the two community’s decisions are

(8) $$U_{Y}=yU_{Y_{1}}+(1-y)U_{Y_{2}}$$

According to Equation (8), the replicator dynamics equation of community groups can be calculated as

(9) $$F(y)=\frac{dy}{dt}=y(1-y)(U_{Y_{1}}-U_{Y_{2}})=y(1-y)[x(G_{Y}^{X}+F_{Y})+z(C_{Y}^{Z})-C_{Y}-C_{Y}^{Z}-L_{Y}]$$

To further analyze the impact of the size of community groups with different strategies on the stable equilibrium of community’s strategy evolution, we can obtain the derivative of the replicator dynamics equation with respect to *y* as follows:

(10) $$\frac{dF(y)}{dy}=(1-2y)[x(G_{Y}^{X}+F_{Y})+z(C_{Y}^{Z})-C_{Y}-C_{Y}^{Z}-L_{Y}]$$

For communities, we can make the following summary according to Equation (10):

(1) When $\frac{C_{Y}+C_{Y}^{Z}+L_{Y}-zC_{Y}^{Z}}{G_{Y}^{X}+F_{Y}}<x\leq 1$, $\frac{dF(y)}{dy}{|}_{y=0}>0$ and $\frac{dF(y)}{dy}{|}_{y=1}<0$, the evolutionary stability strategy (ESS) of the community group is $y^{*}=1$. In this scenario, when the proportion of governments that choose “high-intensity regulation” strategy x is high, the community group tends to choose the “strengthen community prevention and control” $Y_{1}$.

(2) When $x=\frac{C_{Y}+C_{Y}^{Z}+L_{Y}-zC_{Y}^{Z}}{G_{Y}^{X}+F_{Y}}$, F(y)≡0, in this scenario, any proportion *y* that the community chooses “strengthen community prevention and control” strategy is an evolutionary stable strategy.

(3) When $0\leq x<\frac{C_{Y}+C_{Y}^{Z}+L_{Y}-zC_{Y}^{Z}}{G_{Y}^{X}+F_{Y}}$, $\frac{dF(y)}{dy}{|}_{y=1}>0$ and $\frac{dF(y)}{dy}{|}_{y=0}<0$, the evolutionary stability strategy (ESS) of the community group is $y^{*}=0$. In this scenario, when the proportion of governments that choose “high-intensity regulation” strategy *x* is low, the community group tends to choose the “do not strengthen community prevention and control” $Y_{2}$.

According to the above analysis and referring to Figure 1, the feasible area of each game subject is divided into two adjacent areas through the intersection space of x and z. When the feasible region is located in space $v_{3}$, *y* converges to $y^{*}=1$, and it is the optimal decision for the community group to adopt “strengthen community prevention and control” strategy. When the feasible region is located in space $v_{4}$, *y* converges to $y^{*}=0$, and it is the optimal decision for the community group to adopt “do not strengthen community prevention and control” strategy.

#### 3.2.3. Analysis of the Resident’s Evolutionary Stability Strategy

According to the assumptions in the previous section, the probability of the resident choosing $Z_{1}$ (Cooperate with epidemic prevention work) strategy is y, and the probability of choosing $Z_{2}$ (Do not cooperate with epidemic prevention work) strategy is (1−z). $U_{Z_{1}}$ and $U_{Z_{2}}$ are used to represent the expected benefit of the group that the resident chooses $Z_{1}$ and $Z_{2}$, respectively, and represent the overall expected benefit of the resident, so:

The expected benefits when the resident chooses “cooperate with epidemic prevention work” strategy are

(11) $$\begin{array}{l} U_{Z_{1}}=xy(-C_{Z}-C_{Z}^{Y}+A_{Z}^{X}+H_{Z}^{Y}+H_{Z}^{X})+x(1-y)(-C_{Z}+A_{Z}^{X}+H_{Z}^{X})\\ +(1-x)y(-C_{Z}-C_{Z}^{Y}+H_{Z}^{Y})+(1-x)(1-y)(-C_{Z}) \end{array}$$

The expected benefits when the resident chooses “do not cooperate with epidemic prevention work” strategy are

(12) $$U_{Z_{2}}=xy(-F_{Z}+H_{Z}^{Y}+H_{Z}^{X})+x(1-y)(-F_{Z}+H_{Z}^{X})+(1-x)y(H_{Z}^{Y})$$

According to Equations (11) and (12), the overall expected benefits of the two resident’s decisions are

(13) $$U_{Z}=zU_{Z_{1}}+(1-z)U_{Z_{2}}$$

According to Equation (12), the replicator dynamics equation of resident groups can be calculated as

(14) $$F(z)=\frac{dz}{dt}=z(1-z)(U_{Z_{1}}-U_{Z_{2}})=z(1-z)[x(A_{Z}^{X}+F_{Z})+y(-C_{Z}^{Y})-C_{Z}]$$

To further analyze the impact of the size of resident groups with different strategies on the stable equilibrium of resident’s strategy evolution, we can obtain the derivative of the replicator dynamics equation with respect to *z*:

(15) $$\frac{dF(z)}{dz}=(1-2z)[x(A_{Z}^{X}+F_{Z})+y(-C_{Z}^{Y})-C_{Z}]$$

For residents, we can make the following summary according to Equation (15):

(1) When $\frac{yC_{Z}^{Y}+C_{Z}}{A_{Z}^{X}+F_{Z}}<x\leq 1$, $\frac{dF(z)}{dz}{|}_{z=0}>0$ and $\frac{dF(z)}{dz}{|}_{z=1}<0$, the evolutionary stability strategy (ESS) of the resident group is $z^{*}=1$. In this scenario, when the proportion of governments that choose “high-intensity regulation” strategy x is high, the resident group tends to choose the “cooperate with epidemic prevention work” strategy $Z_{1}$.

(2) When $x=\frac{yC_{Z}^{Y}+C_{Z}}{A_{Z}^{X}+F_{Z}}$, F(z)≡0, in this scenario, any proportion *z* that the resident chooses “cooperate with epidemic prevention work” strategy is an evolutionary stable strategy.

(3) When $0\leq x<\frac{yC_{Z}^{Y}+C_{Z}}{A_{Z}^{X}+F_{Z}}$, $\frac{dF(z)}{dz}{|}_{z=1}>0$ and $\frac{dF(z)}{dz}{|}_{z=0}<0$, the evolutionary stability strategy (ESS) of the resident group is $z^{*}=0$. In this scenario, when the proportion of governments that choose “high-intensity regulation” strategy *x* is low, the resident group tends to choose the “do not cooperate with epidemic prevention work” $Z_{2}$.

According to the above analysis and referring to Figure 1, the feasible area of each game subject is divided into two adjacent areas through the intersection space of x and y. When the feasible region is located in space $v_{5}$, *z* converges to $z^{*}=1$, and it is the optimal decision for the resident group to adopt “cooperate with epidemic prevention work” strategy. When the feasible region is located in space $v_{6}$, *z* converges to $z^{*}=0$, and it is the optimal decision for the resident group to adopt “do not cooperate with epidemic prevention work” strategy.

According to the above stability analysis, we can get the possible stable equilibrium states under different space combinations, as shown in Table 3. For example, the symbol “$v_{4}(y\to 0)$“ in Table 3 indicates that when the feasible region is $v_{4}$, *y* in the feasible region converges to 0. Therefore, in the spatial combination $\left(v_{1},v_{4},v_{6}\right)$, the stable equilibrium strategy of the evolutionary game converges to (1,0,0). In this case, all government departments have adopted high-intensity regulatory strategies, and all communities and residents have chosen negative prevention and control strategies. However, this stable state of convergence is not the ideal state that the government hopes to achieve. For the government, the ideal stable state should be the result of the combination of $\left(v_{1},v_{3},v_{5}\right)$ spaces, which is (1,1,1). The government takes high-intensity regulations, while all communities and residents actively participate in the epidemic prevention and control work. However, whether the system can achieve such a stable equilibrium strategy largely depends on the model’s initial conditions, such as changes in key parameters like government penalties, government assistance, and additional publicity costs. Therefore, verifying the correctness and effectiveness of the game model through simulation experiment analysis is of great significance for exploring the impact of main variables on the enthusiasm of community and residents’ epidemic control and the effectiveness of national epidemic control.

## 4. Design and Analysis of Reward and Punishment Mechanism

**Figure 2:**
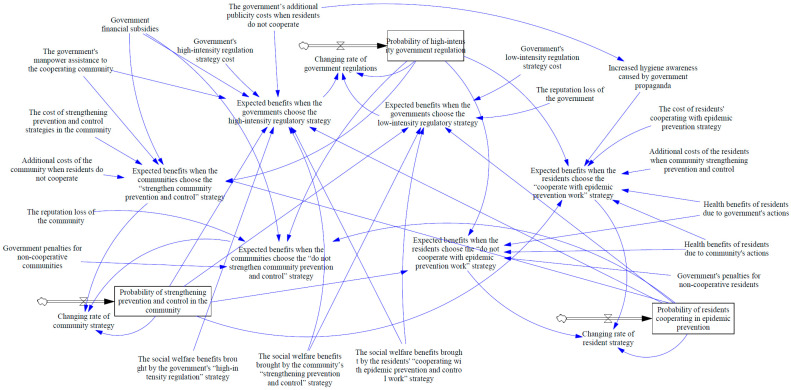
System dynamics stock-flow diagram of government departments, communities, and residents.

**Figure 3:**
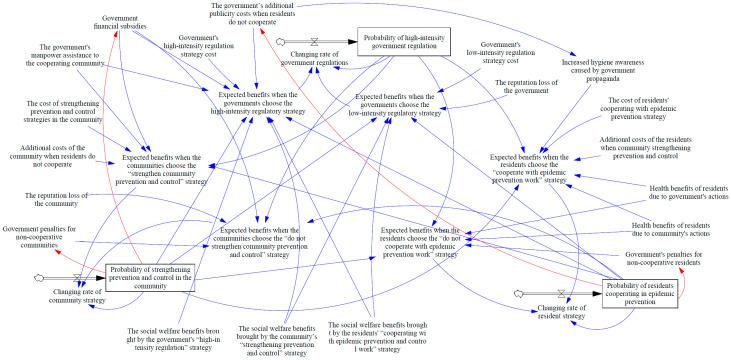
System dynamics stock-flow diagram of government departments, communities, and residents under the dynamic reward and punishment mechanism.

The governments, communities, and residents need to invest many human and material resources to prevent and control the epidemic. Therefore, all participants will lack the motivation to take active prevention and control decisions voluntarily. This situation will hinder the maximization of social welfare and the optimal allocation of resources among all participants. Therefore, the government needs to take appropriate reward and punishment measures to guide the community and residents, which can not only inhibit the free-riding behavior of the participants but also encourage the entire society to take more active measures to improve the overall welfare. Therefore, in this article, we established a mechanism for static rewards and punishments and a mechanism for dynamic rewards and punishments, as shown in Figure 2 and Figure 3.

System dynamics is a method used to analyze multi-variable nonlinear complex systems. It is built by combining qualitative and quantitative methods based on the variables and their internal linkage in the system. The model of system dynamics includes three types of variables: The level variable is a state variable. It represents the cumulative level of the variable at a certain moment, which can be expressed by the integral of the rate variable; rate variable represents the change rate of the level variable at a certain time; auxiliary variables stand for intermediate variables in the decision-making process, used to describe the information transfer and conversion process between level variables and rate variables. The variables and their relationship constitute a model of system dynamics.

### 4.1. System Dynamics Model under the Static Reward and Punishment Mechanism

This section will study the impact of key parameters on the evolution of the national epidemic management system based on system dynamics modeling and simulation and observe the degree of influence of each parameter on the game’s evolution. In the static reward and punishment mechanism, the enforcement strength of the supervisory department is a fixed value. The rewards and penalties will not change based on changes in the proportion of each participant’s game strategy.

The system dynamics model proposed in this paper is designed for the prevention and control of China’s epidemic situation. It contains three subsystems: the government department subsystem, the community subsystem, and the resident subsystem. The representation of the replication dynamic equation and the relationship between the variables in the evolutionary game model lays the foundation for building the system dynamics model. Therefore, we can draw the system dynamics stock-flow diagram based on the above assumptions (see Figure 2). As a scientific tool for dynamic game process simulation and scenario analysis, the system dynamics model provides a visual representation of the key variables that affect participants’ decision-making in the dynamic system.

Based on Lyapunov functions [35] and Equations (4), (9) and (14), the key equations involved in the system dynamics model can be expressed as

(16) $$X_{i}=x_{0}+\int_{1}^{i}VX_{t}\;dt$$

(17) $$Y_{i}=y_{0}+\int_{1}^{i}VY_{t}\;dt$$

(18) $$Z_{i}=z_{0}+\int_{1}^{i}VZ_{t}\;dt$$

(19) $$x_{i}=X_{i}/(X_{i}+NX_{i})$$

(20) $$y_{i}=Y_{i}/(Y_{i}+NY_{i})$$

(21) $$z_{i}=Z_{i}/(Z_{i}+NZ_{i})$$

(22) $$VX_{i}=dx_{i}/dt=x_{i}(1-x_{i})(UX_{i}-UNX_{i})$$

(23) $$VY_{i}=dy_{i}/dt=y_{i}(1-y_{i})(UY_{i}-UNY_{i})$$

(24) $$VZ_{i}=dz_{i}/dt=z_{i}(1-z_{i})(UZ_{i}-UNZ_{i})$$

$X_{i}$, $NX_{i}$, $Y_{i}$, $NY_{i}$, $Z_{i}$, and $NZ_{i}$ respectively represent the ratio of the government’s two strategies, the community’s two strategies, and the residents’ two strategies at time *i*. $x_{0}$, $y_{0}$, and $z_{0}$ respectively represent the initial values of *x*, *y*, and *z*. *VX*, *VY*, and *VZ* respectively represent the rate change of the government’s “high-intensity regulation” strategy, the community’s “strengthening prevention and control” strategy, and the residents’ “cooperate with epidemic prevention work” strategy. *UX*, *UNX*, *UY*, *UNY*, *UZ*, and *UNZ* respectively represent the expected benefits of the government’s two strategies, the expected benefits of the community’s two strategies, and the expected benefits of the residents’ two strategies.

**Figure 4:**
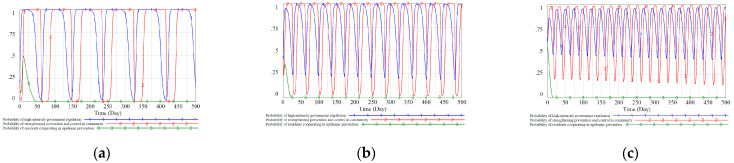
The impact of different initial strategy combinations on the evolutionary stable equilibrium strategy under the static reward and punishment mechanism. (**a**): initial value strategy combinations $\left(x_{0}=0.2,\;y_{0}=0.2,\;z_{0}=0.2\right)$. (**b**): initial value strategy combinations $\left(x_{0}=0.4,\;y_{0}=0.4,\;z_{0}=0.4\right)$. (**c**): initial value strategy combinations $\left(x_{0}=0.6,\;y_{0}=0.6,\;z_{0}=0.6\right)$.

The paper is based on Vensim PLE 8.2.1 for modeling and simulation. The simulation interval is [0,500], where INITIAL TIME = 0, FINAL TIME = 500 Day, TIME STEP = 1 Day, and the integration type is Euler. According to the parameter constraints in the previous section, combined with expert suggestions, the relevant parameters of the tripartite evolutionary game model we proposed are set as follows: $C_{X}^{L}=1$, $C_{X}^{H}=4$, $C_{X}^{Z}=0.7$, $L_{x}=2$, $S_{Y}^{X}=0.6$, $G_{Y}^{X}=1$, $R_{x}=3$, $R_{y}=2$, $R_{z}=1$, $C_{Y}=2.5$, $C_{Y}^{Z}=0.8$, $L_{Y}=1$, $F_{Y}=2$, $A_{Z}^{X}=0.5$, $C_{Z}=0.8$, $C_{Z}^{Y}=0.6$, $H_{Z}^{X}=0.7$, $H_{Z}^{Y}=0.5$, $F_{Z}=0.8$. We refer to the evolutionary game model designed by Zhu et al. [24] and consider three different initial value strategy combinations $\left(x_{0}=0.2,\;y_{0}=0.2,\;z_{0}=0.2\right)$, $\left(x_{0}=0.4,\;y_{0}=0.4,\;z_{0}=0.4\right)$, $\left(x_{0}=0.6,\;y_{0}=0.6,\;z_{0}=0.6\right)$, and the simulation results are shown in Figure 4.

.

According to the evolution process in Figure 4, under the static reward and punishment mechanism, the government cannot mobilize residents’ enthusiasm to cooperate with epidemic control work. In the scenario of different initial value ratio strategy combinations, the ratio of residents “cooperate with epidemic prevention work” stabilized at z=0 after t>50. At the same time, the proportion of government and community strategies is still fluctuating. Comparing Figure 4a–c, it can be seen that the higher the initial decision-making ratio of the three participants, the more intense the decision-making fluctuations. Moreover, there is a relatively fixed periodicity in the fluctuation of the decision-making ratio between the government and the community. The change cycle of the community has a certain delay compared with the change cycle of the government. Community decision-making changes often follow government decision-making changes, and the government has a certain degree of dominance in the process of strategy evolution.

We can see that under the static reward and punishment mechanism, fluctuations in the evolutionary game process between the government and the community have always existed. And the residents’ awareness of active epidemic prevention has not been stimulated. This scenario is common in real life, that is, the negative phenomenon of “government creates policies and community have policy countermeasures”. Residents have a fluke attitude of “the law cannot be enforced when everyone is an offender” to avoid responsibility. During the difficult epidemic situation, government departments will take the lead in adopting high-intensity regulatory strategies to supervise and urge communities and residents effectively. However, as regulatory strategies become more and more effective, some government agencies will reduce their regulatory efforts and relax supervision and subsidies to reduce regulatory costs. Due to the lack of subsidy measures and regulatory policies, the willingness of communities and residents to actively prevent and control the epidemic has been continuously reduced, which has worsened the situation of epidemic control and reduced overall social welfare. Then government agencies had to increase regulation again, which resulted in periodic fluctuations in the ratio of government to community decision-making.

The stability of implementing government regulatory measures is crucial to the overall epidemic prevention and control situation. The continuous adjustment of different government regulatory strategies is not only detrimental to the stability of the epidemic control situation and the long-term effectiveness of the government’s regulatory strategies, but also increases the burden of actual implementation of policies. The cyclical fluctuations in the decision-making ratio of each subject during the game process will cause the government, as the makers of epidemic control policies and the guide of residents’ behavior, to misjudge the effects of the policies. In addition, excessively severe penalties will harm the interests of communities and residents, and excessively mild penalties may not mobilize the enthusiasm of the society for active control and cause the loss of social welfare. Therefore, a good reward and punishment mechanism should meet the needs of the social environment and epidemic control situation in different periods during the implementation process, dynamically adjusting the rewards and punishments promptly. In order to achieve the ideal supervision effect and avoid the oscillation in the evolution of the game. Therefore, based on the game relationship in this section, we further consider the impact of the dynamic reward and punishment mechanism.

### 4.2. System Dynamics Model under Dynamic Reward and Punishment Mechanism

Under the dynamic reward and punishment mechanism, the government considers the proportion of communities and residents in cooperation with epidemic control. It dynamically adjusts the rewards and punishments to guide communities and residents to make decisions that meet the needs of epidemic control. Specifically, when the ratio of cooperation between communities and residents is low, penalties are reduced, and rewards are increased, with rewards as the primary incentive method. When it is higher, the penalties are increased, and the rewards are reduced, with punishment as the primary incentive method.

In terms of subsidy measures, the government will provide a higher intensity of subsidies at the initial stage when the proportion of each participants’ cooperative strategy starts to grow, alleviating the problem of high cold-start costs for each participant’s cooperative strategy. It encourages all entities to actively choose decisions that are conducive to the control of the epidemic. In the mid-late stages of the participants’ cooperation strategies’ proportion increase, the proportion of community and resident’s cooperative strategy is relatively high. The government will no longer blindly use high subsidies to achieve desired goals and appropriately reduce the subsidy intensity to reduce regulation costs.

In terms of punishment measures, at the initial stage when the proportion of each participants’ cooperative strategy start to grow, to avoid the objectively existing fluke attitude of “the law cannot be enforced when everyone is an offender” in each group, the government mainly use subsidy policy to mobilize the enthusiasm of each group to cooperate. Therefore, the punishment measures are relatively weak. In the mid-late stages of the participants’ cooperation strategies’ proportion increase, the proportion of community and resident’s non-cooperative strategy is relatively high. Then, the punishment will be increased and highlight the “vital few” to provide continuous cooperative incentives.

The dynamic reward and punishment parameter can be expressed as

(25) $${F_{Y}}^{*}=yF_{Y}+b_{1}$$

(26) $${F_{Z}}^{*}=zF_{Z}+b_{2}$$

(27) $$S_{Y}^{X^{*}}=(1-y)S_{Y}^{X}+b_{3}$$

(28) $$C_{X}^{Z^{*}}=(1-z)C_{X}^{Z}+b_{4}$$

We use integral constraints to make the government’s dynamic rewards and penalties approximately equal to those in static conditions. For ${F_{Y}}^{*}$, that is $\int_{0}^{1}(yF_{Y}+b_{1})\;dy=F_{Y}$. At this time, when the proportion of the $Y_{1}$ strategy selected by the community is equal to that of the $Y_{2}$ strategy selected, the strength under the dynamic reward and punishment mechanism is equal to those in static conditions. By solving, $b_{1}=1$. Similarly, we can get: $b_{2}=0.4$, $b_{3}=0.3$, $b_{4}=0.35$. The evolutionary game process under the dynamic reward and punishment mechanism is shown in Figure 3.

**Figure 5:**
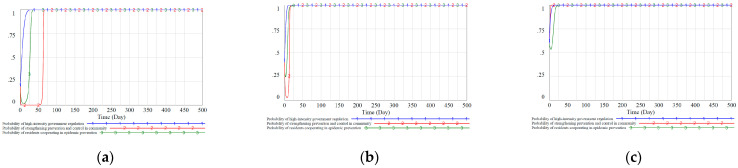
The impact of different initial strategy combinations on the evolutionary stable equilibrium strategy under the dynamic reward and punishment mechanism. (**a**): initial value strategy combinations $\left(x_{0}=0.2,\;y_{0}=0.2,\;z_{0}=0.2\right)$. (**b**): initial value strategy combinations $\left(x_{0}=0.4,\;y_{0}=0.4,\;z_{0}=0.4\right)$. (**c**): initial value strategy combinations $\left(x_{0}=0.6,\;y_{0}=0.6,\;z_{0}=0.6\right)$.

According to the evolution process in Figure 5, we can see that under the dynamic reward and punishment mechanism, the enthusiasm of communities and residents to take the initiative to prevent and control the epidemic has been mobilized. In the scenario of different initial value ratio strategy combinations, the community groups and residents finally made measures conducive to epidemic control. Comparing Figure 4a–c, we can see that compared with the static reward and punishment mechanism, the dynamic mechanism considers the changes in the social environment and epidemic control situation. This can effectively suppress the fluctuation problem in the evolutionary game process under the static reward and punishment mechanism. A stable policy is a prerequisite for social stability, and it will produce better results from the perspective of a long-term mechanism. Based on the above analysis, we can conclude from the perspective of system controllability that the dynamic reward and punishment mechanism is significantly better than the static reward and punishment mechanism.

### 4.3. Simulation Analysis of the Impact of Key Exogenous Variables

#### 4.3.1. The Impact of Government Financial Subsidies to Communities on the Evolutionary Game Process

The government’s financial subsidy to the community is an important strategy to mobilize the community’s enthusiasm for epidemic control in the early stage. In the early stage of the epidemic, the community needed various supplies to implement epidemic prevention and control methods. Due to high economic costs, communities have to adopt passive epidemic prevention control strategies. Therefore, the government’s financial subsidy to the community can promote the community to strengthen their epidemic prevention and control. However, in the same way, excessive fiscal subsidies will cause the government to have a high fiscal deficit, making it difficult for the government to make decisions conducive to epidemic control due to financial pressure. Therefore, reasonable financial subsidies can promote the community to strengthen epidemic control and reduce the government’s fiscal expenditure, making the government more inclined to adopt high-intensity regulatory measures.

**Figure 6:**
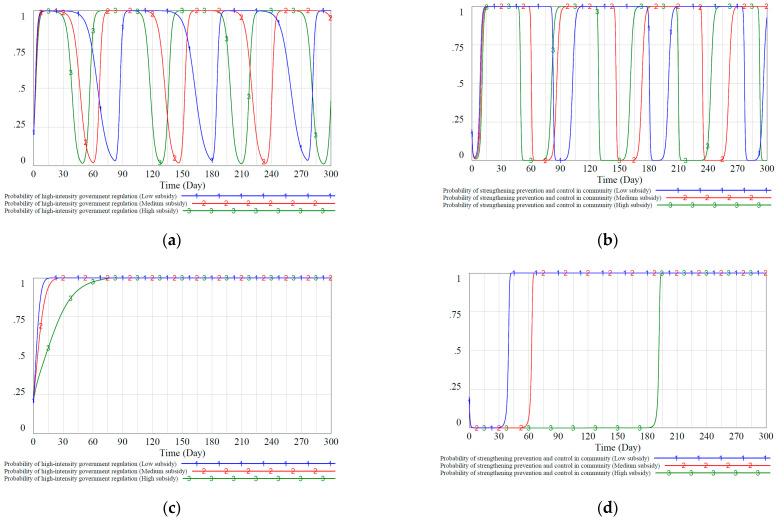
Proportion of the evolutionary stable equilibrium strategy of the government and the community under the static and dynamic reward and punishment mechanisms. (**a**): probability of high-intensity government regulation in different subsidy intensities under the static mechanism. (**b**): probability of strengthening prevention and control in community in different subsidy intensities under the static mechanism. (**c**): probability of high-intensity government regulation in different subsidy intensities under the dynamic mechanism. (**d**): probability of strengthening prevention and control in community in different subsidy intensities under the dynamic mechanism.

We simulated three different government subsidy strategies for communities under static and dynamic reward and punishment mechanisms to study how these variables will affect the evolution of the government’s and community’s strategies. We set the value of the government’s financial subsidy $S_{Y}^{X}$ to the community in the static reward and punishment mechanism to 0.5, 0.6, and 0.7, corresponding to low, medium, and high subsidies, respectively. Similarly, we scale the subsidy intensity in the dynamic reward and punishment mechanism proportionally, and the simulation results are shown in Figure 6.

Figure 6a,b show the evolution of the proportion of government and community decision-making under different subsidies. It can be observed that with the increase of government subsidies to communities, the cycle of decision-making changes between the government and communities has shortened, and the fluctuations have not weakened but increased. It is because when the government subsidies are large, the cost of the government choosing a high-intensity regulatory strategy is relatively high. Out of the rational thinking of maximizing its interests, the government will change its own decisions more quickly to ensure a lower regulatory cost and a higher overall return during the game. By comparing Figure 6c,d, it can be observed that the system stability in the dynamic reward and punishment situation is better than that in the static situation, and the anti-interference is better.

We can see from Figure 6 that the government has a dominant position in the game’s decision-making. As the policymaker and the guide of the society, the government’s decision-making changes often precede the community’s decision-making changes. Specifically, it can be seen from Figure 6d that the proportion of community groups that choose to strengthen community prevention and control first decreased and then increased. The reason is that the early government groups chose a relatively low proportion of high-intensity regulatory strategies. At this time, the government cannot provide sufficient financial support to the community, and community groups have to choose negative strategies due to high prevention and control costs. In addition, it can be observed from Figure 6c,d. In the dynamic reward and punishment mechanism, when the government’s subsidies to the community increase, the speed of the government group reach stable equilibrium slows down due to higher regulatory costs. At this time, the speed of the community reaches stable equilibrium strategy slows down. Therefore, the government should give full play to its leading role, take the lead in adopting high-intensity regulatory strategies during the epidemic, and set appropriate community subsidies to guide communities to actively start epidemic prevention and control work.

#### 4.3.2. The Impact of Government Punishment on the Evolutionary Game Process

Government punishment is also an important measure to guide communities and residents to make positive strategies. In theory, if the government’s punishment for not cooperating with communities or residents expanded indefinitely, it can ensure that all communities and residents adopt strategies that meet the government’s expectations. However, punishment is not the ultimate goal of the government. The expected goal of the government is to create a healthy and stable society by mobilizing the enthusiasm of the community and residents to take the initiative to prevent the epidemic. Excessive penalties will significantly damage the interests of communities and residents and cause social unrest. Therefore, appropriate penalties can not only serve as a warning, and guide communities and residents to take the initiative to prevent the epidemic. It is also possible to highlight the “vital few” that do not cooperate.

**Figure 7:**
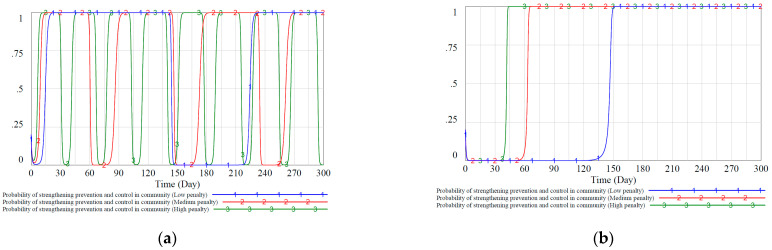
Proportion of the evolutionary stable equilibrium strategy of the community under the static and dynamic reward and punishment mechanisms. (**a**): probability of strengthening prevention and control in community in different penalty intensities under the static mechanism. (**b**): probability of strengthening prevention and control in community in different penalty intensities under the dynamic mechanism.

We simulated three different government penalties for communities and residents under static and dynamic reward and punishment mechanisms to study how these variables will affect the evolution of the community’s and residents’ strategies. Regarding the government’s punishment to the community, we set the value of the government’s punishment F to the community in the static reward and punishment mechanism as 1.4, 2, and 2.6, respectively, corresponding to lower, medium, and higher penalties. Similarly, we scale the penalties intensity in the dynamic reward and punishment mechanism proportionally, and the simulation results are shown in Figure 7.

Figure 7a shows that when the government increases penalties on the community in a static situation, the cycle of community decision-making changes shortens, and the fluctuations increase. Figure 7a shows that when the government increases penalties on the community in a static situation, the cycle of community decision-making changes shortens, and the fluctuations have not weakened but increased. This is because when the government penalties are large, the cost of the community choosing not to strengthen community prevention and control strategy is relatively high. Out of the rational thinking of maximizing its interests, the community will change its decisions more quickly to ensure a lower regulatory cost and a higher overall benefit during the game. Figure 7b shows that when the government increases the punishment of the community under the dynamic reward and punishment mechanism, the community adopts the strategy of “strengthening community prevention and control” faster, which shows that the community reacts positively to the punishment. In addition, it can be observed that the system stability of community strategy evolution in the dynamic reward and punishment mechanism is also better than the static mechanism, and its anti-interference is better.

**Figure 8:**
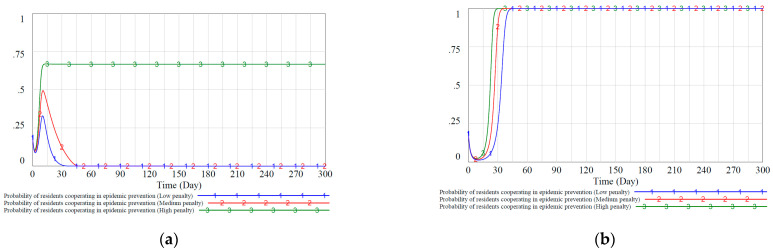
Proportion of residents’ evolutionary stable equilibrium strategies under static and dynamic punishment scenarios. (**a**): probability of residents cooperating in epidemic prevention in different penalty intensities under the static mechanism. (**b**): probability of residents cooperating in epidemic prevention in different penalty intensities under the dynamic mechanism.

Regarding the government’s punishment to residents, we set the value of the government’s punishment $F_{Z}$ to residents in the static reward and punishment mechanism as 0.7, 0.8, and 0.9, corresponding to lower, medium, and higher penalties, respectively. Similarly, we scale the government’s penalties to residents in the dynamic reward and punishment mechanism proportionally, and the simulation results are shown in Figure 8.

Figure 8a shows that residents will react more sensitively when the government increases or reduces the punishment to residents in the static reward and punishment mechanism. Reducing the intensity of penalties will enable residents to achieve a stable strategy of negative prevention and control faster due to insufficient penalties. Slightly higher penalties will appropriately increase residents’ enthusiasm for active epidemic prevention so that a certain proportion of residents are stabilized in the active epidemic prevention strategy $X_{1}$ (about 0.65). However, the effect is still worse than the dynamic reward and punishment mechanism in Figure 8b. Figure 8b shows that when the government’s punishment to residents increases, the residents will adopt the active epidemic prevention strategy more quickly, indicating that residents are active in responding to punishment. In addition, it can be observed that the system stability of resident strategy evolution in the dynamic reward and punishment mechanism is also better than the static mechanism, and its anti-interference is better.

Figure 7a and Figure 8a show that in the static punishment mechanism with different punishment intensities, the communities and residents still appear the same situation as shown in Figure 6. That is, the proportion of community groups who choose the “strengthen community prevention and control” strategy and the proportion of residents who choose the “cooperate with epidemic prevention and control” strategy first decrease and then increase. In the dynamic penalty scenarios shown in Figure 7b and Figure 8b, although the penalty intensity was dynamically adjusted to a smaller level in the early stage when the proportion increased, there was also a trend of decline first and then rise. This shows that in addition to subsidy measures, if the government hopes that the punishment measures have a better implementation effect, it also needs first to mobilize its enthusiasm for implementing the “high-intensity regulation” strategy.

#### 4.3.3. The Impact of Government Propaganda Measures on the Evolutionary Game Process

The contemporary information revolution is developing rapidly, and government information disclosure and public opinion guidance play an important role in national governance. Through health education and publicity, government departments can urge residents to pay attention to epidemic prevention, advocate residents’ awareness and behavior of social responsibility to prevent and control infectious diseases, and guide residents to develop good hygiene habits and lifestyles. But in the same way, excessive publicity investment will cause a high fiscal deficit for the government, making it difficult for the government to make decisions conducive to epidemic control due to financial pressure. Therefore, appropriate propaganda intensity can effectively guide residents to cooperate with epidemic control and reduce government fiscal expenditures, making the government more inclined to adopt high-intensity regulatory measures.

**Figure 9:**
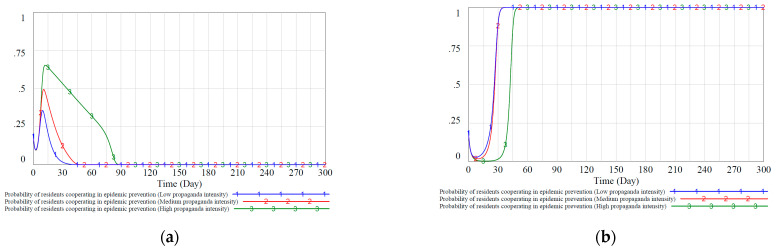
Proportion of residents’ evolutionary stable equilibrium strategies under static and dynamic punishment scenarios. (**a**): probability of residents cooperating in epidemic prevention in different propaganda intensities under the static mechanism. (**b**): probability of residents cooperating in epidemic prevention in different propaganda intensities under the dynamic mechanism.

Therefore, we simulated three different government propaganda strategies under static and dynamic reward and punishment scenarios to study how these variables will affect the evolution of the governments’ and residents’ strategies. We set the government’s publicity cost value $C_{X}^{Z}$ to residents in the static reward and punishment situation as 0.6, 0.7, and 0.8, corresponding to low, medium, and high propaganda intensity, respectively. Similarly, we scale the propaganda intensity under the dynamic reward and punishment scenario in an equal proportion. Assuming that the residents’ acceptance $\alpha_{Z}^{X}$ of propaganda remains unchanged, the simulation results are shown in Figure 9 below.

Figure 9a shows that residents will react sensitively when the government increases or reduces its publicity to residents in the static reward and punishment situation. Reducing the intensity of publicity will enable the residents to achieve a stable strategy of negative epidemic control strategy faster due to lack of health awareness. Slightly higher propaganda intensity will appropriately increase residents’ enthusiasm for active prevention and control, making the ratio of about 0.65 residents choose to cooperate with the decision-making of epidemic prevention. However, this effect is still worse than the dynamic reward and punishment scenario in Figure 9b, and the ratio gradually decreases after reaching the highest point. Figure 9b shows that when the government’s propaganda to residents increases, the speed at which residents adopt active prevention and control strategies slows down, indicating that excessively high propaganda costs also make the government more hesitant to choose high-intensity regulatory strategies.

## 5. Conclusions and Policy Implications

This paper uses the method of combining evolutionary game and system dynamics to study the emergency management of major public health emergencies involving multi-agent participation. This paper discusses the impact of the static reward and punishment scheme and the dynamic reward and punishment scheme on the system’s stability through the simulation analysis of the evolutionary game process. Then we carried out an in-depth scenario analysis based on the static and dynamic reward and punishment mechanisms to study the impact of key parameters on the evolution of each participant’s strategy. The results can be summarized as follows:

(1) The dynamic reward and punishment mechanism introduced in this article can effectively suppress decision-making fluctuations and stabilize the system. This result is similar to the dynamic scheme proposed in the previous study [24,36]. The main reason why dynamic rewards and punishments can suppress fluctuations is that dynamic rewards and punishments can meet the needs of the social environment and epidemic control situations in different periods, and dynamically adjust the intensity promptly, which greatly reduces the opportunistic behavior of each game subject.

(2) Under the static reward and punishment scheme, a slight increase in each entity’s decision-making cost will shorten the entity’s decision-making change cycle, and the fluctuation will not be weakened but intensified. Therefore, as the guide of the society and the makers of policy, the government is not advisable to restrict other game subjects by merely raising or lowering rewards and punishments, and it may be counterproductive.

(3) Whether under the static reward and punishment scheme or the dynamic reward and punishment scheme, the change in the proportion of government decision-making often precedes the change in the proportion of community and residents’ decision-making. Communities and residents will respond based on the government’s decision-making, and the government will also formulate the next phase of strategies based on the community and residents’ feedback. Therefore, the government should give full play to its leading role, take the initiative to share the burden of epidemic prevention and control costs between communities and residents during the epidemic, and set appropriate subsidies and punishments to guide communities and residents to take epidemic control measures actively.

Therefore, the government should pay attention to the following aspects in its decision-making process in the background of epidemic prevention and control:

(1) The government should set up sound subsidy-punishment types to cover all spheres of the community and residents’ life in various ways. For example, at the beginning of the outbreak, the community did not have enough emergency supplies, did not have enough staffing, and had not received training in epidemic prevention management skills. At this time, the government only used a single financial subsidy to encourage the community to have an unsatisfactory effect. The government should thoroughly analyze the difficulties in the epidemic control work in the community and provide targeted subsidies to the community regarding financial allocation, material allocation, personnel assistance, and management training.

(2) Establishing a subsidy-penalty dynamic adjustment mechanism that is more adaptable to the epidemic control situation can generate stable and continuous incentives for communities and residents in all periods of epidemic control. In response to the objective existence of opportunistic behaviors such as “government creates policies and community have policy countermeasures,” “free-riding,” and “the law cannot be enforced when everyone is an offender” that exist in communities and residents, the government needs to adjust subsidies and penalties in a targeted manner dynamically.

For example, in the early stage of the outbreak, most communities and residents did not have the ability to take active measures to prevent or control the epidemic. Instead of using excessive penalties to force residents and communities to cooperate with the government’s epidemic prevention work, the government should adopt a universal and diverse subsidy policy to affect as many communities and residents as possible and reduce their epidemic prevention costs to promote active epidemic prevention strategies. When most communities and residents have adopted active prevention and control strategies, the government can appropriately reduce the subsidies to communities and residents. Because each group already has certain relevant supply and safety awareness at this time, governments should pay close attention to the “vital few” to prevent the anti-epidemic enthusiasm of each group from rebounding. The government should use higher-intensity penalties to curb the “vital few.”

(3) The government should speed up its response, promptly introduce relevant epidemic prevention and control policies, disclose the development of the epidemic, and carry out health education and publicity. This paper assumes that once the government adopts a high-intensity regulatory strategy, it can immediately introduce and implement relevant policies to direct various departments to carry out emergency management. However, under actual circumstances, it takes much time for the government to formulate scientifically reasonable policies that closely integrate the current situation and future development expectations. When a major public health event breaks out, the early time is very precious, and the government will miss the best time for epidemic control if it does not pay attention to it. Therefore, when the government formulates prevention and control strategies, it must condense all the intelligence and resources and give full play to expert think tanks in universities and research institutions. Every decision-making must refer to the research results of experts and think tank teams, then form decision-making judgments.

## Figures and Tables

**Table 1:** Related parameters and definition.

Parameter	Definition
$C_{X}^{L}$	Government’s low-intensity regulation strategy cost
$C_{X}^{H}$	Government’s high-intensity regulation strategy cost
$C_{Y}$	The cost of strengthening prevention and control strategies in the community
$C_{Z}$	The cost of residents’ cooperating with epidemic prevention strategy
$R_{X}$	The social welfare benefits brought by the government’s “high-intensity regulation” strategy
$R_{Y}$	The social welfare benefits brought by the community’s “strengthening prevention and control” strategy
$R_{Z}$	The social welfare benefits brought by the residents’ “cooperating with epidemic prevention and control work” strategy
$G_{Y}^{X}$	The government’s manpower assistance to the cooperating community
$L_{X}$	The reputation loss of the government not adopting high-strength regulatory measures during the epidemic
$L_{Y}$	The reputation loss of the community not strengthening community prevention and control during the epidemic
$C_{X}^{Z}$	The government’s additional publicity costs when residents do not cooperate
$S_{Y}^{X}$	Government financial subsidies to communities
$F_{Y}$	Government penalties for non-cooperative communities
$F_{Z}$	Government’s penalties for non-cooperative residents
$C_{Y}^{Z}$	Additional costs of the community when residents do not cooperate
$C_{Z}^{Y}$	Additional costs of the residents when community strengthening prevention and control
$\alpha_{Z}^{X}$	Residents’ acceptance to propaganda measures
$A_{Z}^{X}$	Increased hygiene awareness caused by government propaganda, $A_{Z}^{X}=\alpha_{Z}^{X}C_{X}^{Z}$
$H_{Z}^{X}$	Health benefits of residents due to government’s actions
$H_{Z}^{Y}$	Health benefits of residents due to community’s actions

**Table 2:** Game payment matrix of government, community, and resident.

**Government Strategy:** $X_{1}$		
	Resident strategy: $Z_{1}$	Resident strategy: $Z_{2}$
community strategy: $Y_{1}$	$-C_{X}^{H}+R_{X}+R_{Y}+R_{Z}-G_{Y}^{X}-S_{Y}^{X}$ $-C_{Y}+G_{Y}^{X}+S_{Y}^{X}$ $-C_{Z}-C_{Z}^{Y}+A_{Z}^{X}+H_{Z}^{Y}+H_{Z}^{X}$	$-C_{X}^{H}+R_{X}+R_{Y}-G_{Y}^{X}-C_{X}^{Z}-S_{Y}^{X}$ $-C_{Y}+G_{Y}^{X}-C_{Y}^{Z}+S_{Y}^{X}$ $-F_{Z}+H_{Z}^{Y}+H_{Z}^{X}$
community strategy: $Y_{2}$	$-C_{X}^{H}+R_{X}+R_{Z}-S_{Y}^{X}$ $-F_{Y}+S_{Y}^{X}-L_{Y}$ $-C_{Z}+A_{Z}^{X}+H_{Z}^{X}$	$-C_{X}^{H}+R_{X}-C_{X}^{Z}-S_{Y}^{X}$ $-F_{Y}+S_{Y}^{X}-L_{Y}$ $-F_{Z}+H_{Z}^{X}$
**Government Strategy:** $X_{2}$		
	Resident strategy: $Z_{1}$	Resident strategy: $Z_{2}$
community strategy: $Y_{1}$	$-C_{X}^{L}+R_{Y}+R_{Z}-L_{X}$ $-C_{Y}$ $-C_{Z}-C_{Z}^{Y}+H_{Z}^{Y}$	$-C_{X}^{L}+R_{Y}-L_{X}$ $-C_{Y}-C_{Y}^{Z}$ $H_{Z}^{Y}$
community strategy: $Y_{2}$	$-C_{X}^{L}+R_{Z}-L_{X}$ $-L_{Y}$ $-C_{Z}$	$-C_{X}^{L}-L_{X}$ $-L_{Y}$ 0

**Table 3:** Evolutionary stable strategies for feasible regions under each combination.

**Feasible Regions**	$v_{1}(x\to 1)$		$v_{2}(x\to 0)$	
	$v_{3}(y\to 1)$	$v_{4}(y\to 0)$	$v_{3}(y\to 1)$	$v_{4}(y\to 0)$
$v_{5}(z\to 1)$	(1,1,1)	(1,0,1)	(0,1,1)	(0,0,1)
$v_{6}(z\to 0)$	(1,1,0)	(1,0,0)	(0,1,0)	(0,0,0)
